# Validation of the Brief Autism Mealtime Behavior Inventory in Parents of Children in Cyprus

**DOI:** 10.3390/children12081067

**Published:** 2025-08-14

**Authors:** Andri Papaleontiou, Vassiliki Siafaka, Louiza Voniati, Alexandros Gryparis, Rafaella Georgiou, Dionysios Tafiadis

**Affiliations:** 1 Department of Speech & Language Therapy, School of Health Sciences, University of Ioannina, 45500 Ioannina, Greece; siafaka@uoi.gr (V.S.); alexandros@uoi.gr (A.G.); r.georgiou@uoi.gr (R.G.); tafiadis@uoi.gr (D.T.); 2 Department of Health Sciences, Speech and Language Therapy, European University, Nicosia 2404, Cyprus; l.voniati@euc.ac.cy

**Keywords:** autism spectrum disorder (ASD), BAMBI, feeding challenges, food selectivity and refusal, parent-reported measure, psychometric validation

## Abstract

**Background/Objectives:** Autism Spectrum Disorder (ASD) includes significant feeding difficulties, behavioral issues, and communication deficits that are linked to serious medical complications and developmental challenges. The Brief Autism Mealtime Behavior Inventory (BAMBI) is a commonly used tool to screen for mealtime behavior problems in children with ASD; however, it lacks validation for use within the Greek-Cypriot population. The current study sought to present the translation, cultural adaptation, and validation of the BAMBI for Greek-Cypriot parents of children with ASD. **Methods:** Three bilingual experts translated the inventory into Greek, following the translation guidelines by the World Health Organization. The inventory was then administered to 117 parents: 42 children with ASD and 75 typically developing children. Principal Component Analysis was used to obtain the tool’s statistical reliability and validity. **Results:** BAMBI-Gr demonstrated strong internal consistency, as indicated by a Cronbach’s alpha of 0.755, and showed excellent test–retest reliability, with an intraclass correlation coefficient of 0.999. PCA identified three key factors: General Refusals, Refusing Food, and Autism-Related Features. Significant differences in BAMBI-Gr scores of the comparative group of parents of children with ASD and parents of typically developing children highlighted the tool’s sensitivity in detecting mealtime behavior problems. Receiver Operating Characteristics analysis set the cut-off points for optimum distinguishing of feeding problems at 46.00 (sensitivity 0.738, 1-specificity 0.000). **Conclusions:** The Greek-translated version of the BAMBI demonstrates validity and effectiveness as a parent-reported assessment tool for identifying feeding and mealtime difficulties in children with ASD.

## 1. Introduction

Autism Spectrum Disorder (ASD) is a neurodevelopmental condition characterized by a wide range of symptoms and challenges that vary in type and severity [1,2]. The DSM-5 (2013) classifies this group mainly as having communication difficulties, behavioral complications, social interaction weaknesses, feeding disorders, and sensory integration conditions, combined with repetitive and stereotyped manners [3,4,5]. As a result, children with ASD face serious lifelong difficulties in the school, family, and social environment [6,7].

Among the challenging behaviors exhibited by ASD children like self-injury, severe temper tantrums, aggression, toileting complications, and sleep disturbances, feeding problems are also observed [8]. Feeding is one of the most fundamental and uncomplicated human processes, required for child growth, development, and health. Chronic feeding difficulties have serious consequences including malnutrition, growth retardation, and the need for medical interventions such as feeding tubes. Feeding difficulties can also affect a child’s cognitive, social, and emotional development, as well as possibly their family relationships and quality of life [9,10,11]. Additionally, a large percentage of children classified as ASD are faced with serious feeding difficulties such as awareness of the quality of food, refusal of many liquid drinks, and particular selectivity towards certain foods, thus manifesting non-habitual feeding behaviors [12,13]. In addition to food selectivity and refusal, other atypical feeding behaviors such as pica—the compulsive eating of non-nutritive substances—and regurgitation (also referred to as rumination) have been observed among children with ASD. Pica is notably more prevalent in children with developmental disorders, including ASD, and may pose serious health risks such as gastrointestinal obstruction, poisoning, and parasitic infections [14,15].

Regurgitation, or the repeated bringing up of food into the mouth, is another concern and is sometimes linked to behavioral patterns or gastrointestinal discomfort. This behavior can lead to esophagitis, malnutrition, and social challenges [16,17]. These feeding-related issues highlight the need for comprehensive behavioral and medical assessment in the care and intervention planning for children with ASD. Based on many references through research, a significant increase in the prevalence of food selectivity and refusal was found to be the most common type of problematic eating behavior assessed among children with ASD, concluding that eating difficulties are frequent problems presented by children with ASD [16,18,19]. Many of these feeding difficulties stem from characteristics that are common in children with autism, such as heightened sensory sensitivities, repetitive behaviors, and a strong preference for routine and sameness. These traits can make certain textures, tastes, or changes in food particularly distressing. Additionally, difficulties with communication and emotional regulation can add to the challenge, making mealtimes especially complex for both children and their families. Given the high prevalence of these challenges, nutritional interventions and specialty diets are often considered part of a broader management approach. Emerging research also highlights the role of the gut–brain axis, suggesting that gastrointestinal health may influence neurological functioning. For instance, the small intestine contributes to the production of neurotransmitters like serotonin, which are involved in regulating mood, behavior, and cognition. These findings underscore the critical need for multidimensional assessment and evidence-based care strategies for children with ASD [20].

It is necessary to assess these feeding challenges faced by children with ASD before initiating any intervention. It is also important that healthcare practitioners are more knowledgeable about this topic, and the possible recognition and screening for feeding difficulties in children with ASD should be incorporated into standard assessment protocols [1,21]. As is already known, there is a variety of methods, including checklists, questionnaires, interviews, and surveys that parents or other caregivers fill out, to assess and determine the variables related to feeding difficulties in children with ASD [22,23,24]. One widely distributed questionnaire assessing mealtime behavior problems in children with ASD is the Brief Autism Mealtime Behavior Inventory (BAMBI) [25,25].

The initial version of BAMBI was designed solely to measure feeding problems and behaviors during the feeding procedure of children with ASD [25]. According to Lukens (2002), the BABMI in its primary form consisted of 20 questions, divided into (a) Food Refusal/Disruptive Behavior factor, (b) Limited Variety factor, and another factor that has not been named or was not evaluated initially [25,25]. Through the implementation of this inventory, these researchers identified the weaknesses of the questionnaire and judged its non-validity, thus updating its new version six years later [25]. The revised questionnaire consists of 18 items and utilizes a Likert scale to measure the frequency of specific mealtime behaviors [25]. This scale scores these behaviors, with 1 = never to 5 = at almost every meal [25]. The BAMBI is characterized as a reliable and accurate instrument for the evaluation and quantification of feeding challenges in typically developing children and those diagnosed with ASD, across several populations. Specifically, it has been translated, with its validity and reliability confirmed, in Italian, Brazilian Portuguese, and Turkish languages [21,26,27]. This outcome highlights the significance and efficacy of the BAMBI’s assessment in addressing feeding challenges and behaviors observed among individuals affected by ASD. Moreover, it underscores the ability of interdisciplinary teams to promptly and effectively implement the necessary interventions, thereby facilitating the achievement of the desired feeding outcomes.

Nevertheless, in the Greek-Cypriot population, there are no validated screening questionnaires for feeding problems in the pediatric population with ASD. Thus, the present study aims to evaluate the psychometric validity of the BAMBI questionnaire in a Greek population, as reported by Greek-Cypriot parents of children diagnosed with ASD who exhibit feeding difficulties.

## 2. Materials and Methods

### 2.1. Instrument

The BAMBI is an assessment tool developed in English to gather observer-reported outcome information on diet and eating/mealtime behaviors from parents and primary caregivers of children with ASD [25,25]. This questionnaire proved to have strong potential for practical use in clinical settings, enabling a straightforward and effective assessment of feeding difficulties in children diagnosed with ASD. The BAMBI has 18 items, determined based on ratings using a five-point Likert scale, beginning at 1  =  never/rarely to 5  =  at almost every meal. The questionnaire also has a reverse scoring system that applies to four questions evaluating positive feeding interactions. The total score is computed from all 18 items, with higher values indicating increased mealtime behavior difficulties, while exploratory factor analysis revealed three distinct factors: Limited Variety, Food Refusal, and Features of Autism [25]. Its primary objective is to reliably and quickly diagnose feeding problems. This identification is advantageous for developing individualized intervention plans to address certain feeding problems, combining nutritional and behavioral therapies to improve the child’s overall dietary intake and eating habits, and facilitating the interdisciplinary exchange of needed information and interventional strategies between clinicians, medical personnel, and parents/caregivers regarding a child’s needs [23,24].

### 2.2. Translation Procedure

After contacting the creators of the BAMBI and requesting authorized approval for translation and validation of this instrument, the questionnaire was linguistically adapted in accordance with WHO methodology (World Health Organization) [28], to preserve semantic, conceptual, and technical integrity from the original tool in the Greek version of the BAMBI. The original questionnaire was independently translated into Greek by three translators proficient in both languages (English and Greek). The translators were SLPs with extensive training in both questionnaire design and the population to be served. The condition mentioned above makes it more likely that the questionnaire will be translated correctly and that the translated text will employ language like that of the target group.

Following this, three bilingual specialists with backgrounds in questionnaire development and healthcare evaluated the translation, searching for discrepancies between the original version and the translated content [28]. The panel of experts deliberated and rectified flaws regarding the protection of the source tool, its semantic integrity, and conceptual and technical equivalency. Another SLP who has expertise in feeding and swallowing disorders, who had not been involved in the original translation of the instrument or consulted the initial version of the questionnaire, conducted a backward translation of the final Greek version of the questionnaire into the source language and cross-referenced it with the original document to ensure that the translation was accurate. Subsequently, in pilot research, the translated questionnaire was evaluated and eventually, the final Greek-translated version of the BAMBI questionnaire was finalized [29].

The translated questionnaire was administered to 117 Greek-Cypriot parents who were responsible for feeding their children. These parents were divided into two groups: (a) the parent group of typically developing (TD) children (N = 75) and (b) the parent group of ASD children (N = 42) who exhibit feeding and swallowing difficulty.

The primary inclusion criterion for the ASD group was a confirmed diagnosis of a feeding and swallowing disorder in the child, assessed by a certified Speech–Language Pathologist (SLP). A confirmed primary diagnosis of autism spectrum disorder (ASD) was required, established by a licensed clinician (psychologist, psychiatrist, or developmental pediatrician) using standardized diagnostic tools, in line with DSM-5 criteria. Additional inclusion criteria included (a) the parent’s physical presence during the re-administration of the questionnaire to ensure consistency in responses, and (b) the child’s age falling between 6 months and 16 years. All participating parents were required to have adequate language proficiency to complete the questionnaire reliably. Children with unrelated comorbid medical conditions that could independently affect feeding behaviors (e.g., genetic syndromes, cerebral palsy) were excluded to control for confounding variables. Participants were selected from different intervention settings in Cyprus, private speech therapy clinics, university clinics for practical intervention, and public and special schools. After receiving signed consent, the research team accessed the participant’s medical records to confirm the diagnosis of feeding and swallowing problems and the official diagnosis of ASD. Each participant completed the translated BAMBI questionnaire, and to evaluate its reliability, all parents were asked to complete it again ten days later.

### 2.3. Ethical Aspects

All participants were informed about the study’s purpose and procedure, as well as about the confidentiality of their personal information, having signed a consent form before enrolment. The research study was authorized by the Cyprus National Bioethics Committee (EEBK/EΠ/2019/95) and the University of Ioannina Bioethics Committee (approval number: 27829).

### 2.4. Statistical Analysis

The statistical analysis involved descriptive characteristics, *t*-tests for independent groups, one-way ANOVA applied to examine variations among several groups, and non-parametric comparisons made using the Kruskal–Wallis’ test. Furthermore, Receiver Operating Characteristic (ROC) curve analysis was utilized to determine the most appropriate threshold determination for the BAMBI. The robust and efficient Youden index was used to determine the ideal threshold values. An alpha value was calculated to assess the internal reliability of the Greek BAMBI. Test–retest reliability was assessed using Pearson’s r coefficient and the Interclass Correlation Coefficient (ICC). Pearson’s r coefficient was used to assess the external criterion validity for the BAMBI questionnaire.

The Content Validity Index (CVI) was used to estimate the validity analysis of the ΒAΜΒΙ in Greek [29]. The Content Validity Index (CVI) was determined based on the evaluations agreed upon by the five speech–language pathologists (SLPs) specializing in pediatric dysphagia. For each item, the Item-Level Content Validity Index (I-CVI) was calculated by dividing the number of speech–language pathologists (*n* = 5) who agreed on the item by the total number of participating experts. In contrast, the total Questionnaire CVI (S-CVI) was obtained by adding the Item CVI and dividing the result by 14. The research states that an I-CVI of 0.78 or higher and an S-CVI of 0.8 are required to classify the questionnaire’s content validity as outstanding [30].

The BAMBI’s 18 items underwent Principal Component Analysis (PCA) to reduce the set of observed variables into a smaller number of latent factors. This statistical technique is commonly used to detect patterns and relationships in multivariate data and reduce its dimensionality. In PCA, original vJamoviariables are transformed into a smaller set of uncorrelated variables known as principal components. Focusing on these components—particularly those that account for the greatest variance—can simplify the analysis and interpretation of questionnaire data.

Prior to conducting PCA, the dataset’s suitability for factor analysis was confirmed using the Kaiser–Meyer–Olkin (KMO) measure of sampling adequacy and Bartlett’s test of sphericity. The number of components to retain was guided by the scree plot and supported by eigenvalues greater than 1.0. Following the identification of the optimal number of components, PCA was carried out, and the scree plot was used to visualize the eigenvalues associated with each component.

Given the moderate sample size (N = 117 families), we adhered to established criteria to ensure reliable factor extraction. Only items with factor loadings above 0.4 were retained to maintain the clarity and interpretability of the factors.

To verify that the data satisfied the assumptions for parametric testing, normality was assessed using both the Kolmogorov–Smirnov and Shapiro–Wilk tests. A two-tailed significance level of *p* < 0.05 was applied. Statistical analyses were performed using the IBM SPSS statistical software (version 28.0, Armonk, NY, USA) and the Jamovi software. [The jamovi project (2023). jamovi. (Version 2.4) [Computer Software] [31,32]. Retrieved from https://www.jamovi.org]. Specifically, for the EFA analysis, the lavaan library in the R package (Version 2.4) was used [33,34].

## 3. Results

### 3.1. Demographic Data and Group Effects

Both groups were comparable regarding age, sex, and educational background. A summary of the demographic information for all participants is provided in Table 1.

With regard to the BAMBI questionnaire total score, the between-group comparisons showed that the parent group of ASD children had statistically significantly higher scores than the parent group of TD children [t (115) = −10.787, *p* < 0.001]. Likewise, statistically distinct patterns emerged within the three factors of the BAMBI questionnaire: (a) for “Limited Refusal”, [t (115) = 4.762, *p* < 0.001], (b) for “Food Refusal”, [t (115) = 12.402, *p* < 0.001], and (c) for “Features of Autism”, [t (115) = 6.528, *p* < 0.001]. Comparisons between groups and the corresponding results are detailed in Table 2.

The main groups’ effect was evaluated following the severity of children with ASD, and the one-way ANOVA method was used. The groups were split into the parent group of level 1 (requiring support) ASD children, the parent group of level 2 (requiring substantial support) ASD children, the parent group of level 3 (requiring very substantial support) ASD children, and the parent group of TD children. The analysis returned the following: for the BAMBI total scores, [F (3, 113) = 40.354, *p* < 0.001]; for the “Limited Refusal” total score, [F (3, 113) = 7.535, *p* < 0.001], for the “Food Refusal” total score, [F (3, 113) = 63.155, *p* < 0.001], and for the “Features of Autism Factor”, total score [F (3, 113) = 13.393, *p* < 0.001], as summarized in Table 3.

The group effect between parents of children with ASD was also explored with the Kruskal–Walli’s test. The analysis indicated no statistically significant differences in BAMBI total scores [H (3) = 1.140, *p* = 0.565], the “Limited Refusal” total score [H (3) = 0.286, *p* = 0.867], the “Food Refusal” total score [H (3) = 5.288, *p* = 0.101], and the “Features of Autism Factor” total score [H (3) = 0.999, *p* = 0.607].

### 3.2. Receiver Operating Characteristics Analysis for the BAMBI

**Figure 1:**
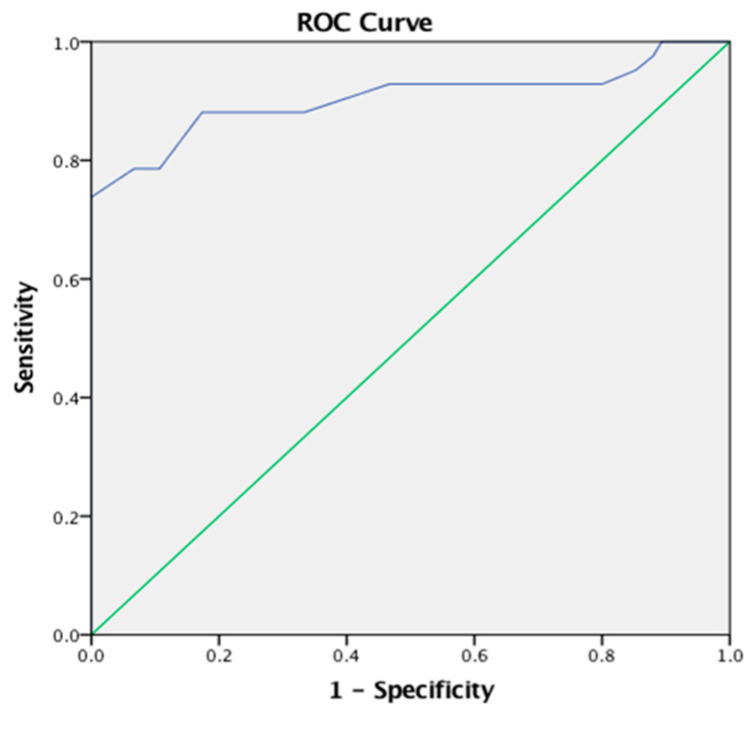
ROC curve comparing BAMBI total scores between parents of children with ASD and parents of typically developing children. The blue line indicates the ROC curve for the BAMBI total score, while the green diagonal line represents the reference line for random classification (AUC = 0.5).

The ROC analysis was conducted to determine the cut-off thresholds for the BAMBI’s total score and its three underlying factors. Notable positive discrimination was identified between the parent group of children with ASD and the parent group of typically developing children in the BAMBI total score [AUC 0.905 (95% CI: 0.833–0.976), *p* < 0.001], with an optimal cut-off point equal to 46.00, a sensitivity of 0.738, and 1-specificity of 0.000 (as shown in Figure 1).

**Figure 2:**
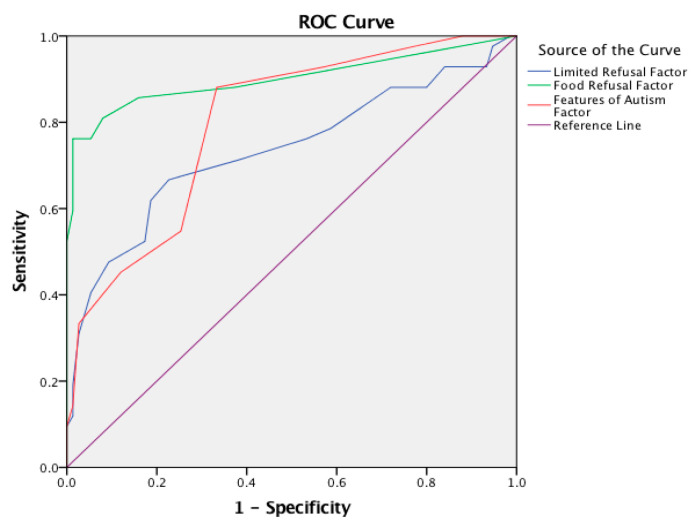
Comparison of BAMBI’s three factors using ROC Curves for parents of ASD and typically developing children.

Likewise, ROC analysis indicated significant positive differentiation among the three factors of the BAMBI: (a) “Limited Refusal” [AUC 0.735 (95% CI: 0.633–0.838), *p* < 0.001], with an optimal cut-off point of 22.00 (sensitivity: 0.667 and 1-specificity: 0.227); (b) “Food Refusal” [AUC 0.900 (95% CI: 0.829–0.972), *p* < 0.001], with an optimal cut-off point of 9.00 (sensitivity: 0.762 and 1-specificity: 0.013); (c) “Features of Autism” [AUC 0.696 (95% CI: 0.629–0.763), *p* < 0.001], with an optimal cut-off point of 12.00 (sensitivity: 0.881 and 1-specificity: 0.333) (as presented in Figure 2).

### 3.3. Reliability and Validity Measures for ΒΑΜΒΙ-Gr

The BAMBI’s internal consistency was very good (Cronbach alpha = 0.755). The item-scale correlations of the BAMBI’s 18 items ranged from 0.725 to 0.781. The test–retest reliability was estimated between the first and the second administration of the BAMBI questionnaire. Specifically, a high degree of reliability was measured for the BAMBI total score, with an ICC equal to 0.999 [95%, CI: 0.999–1.00]. Likewise, the Pearson correlation showed a significantly high degree of reliability r = 0.999 *p* < 0.001.

To establish the content validity of ΒAΜΒΙ in the Greek Language, the S-CVI was computed and was equal to 1. The CVI for all items’ clearance of the questionnaire was equal to 1, and the total agreement was 18 between the evaluators. To assess the BAMBI’s sensitivity to an external validity criterion, we next performed a Pearson’s correlation between the scale’s overall score and its three components and the Montreal Children’s Hospital Feeding Scale (MCH-FS) total score. In particular, the analysis revealed a strong positive correlation (r = 0.780, *p* < 0.001) between the MCH-FS total score and the BAMBI total mean score, as well as positive correlations between the MCH-FS and the “Limited Refusal Factor” total score, the “Food Refusal” total score (r = 0.647, *p* < 0.001), and the “Features of Autism” total score (r = 0.586, *p* < 0.001) (seen in Table 4).

### 3.4. Factor Analysis for the BAMBI

The correlations between the BAMBI questionnaire’s 18 items were examined before conducting PCA. Since the correlations ranged between −0.6 and 0.7, none of the items were excluded. PCA was used to assess the construct validity of the BAMBI questionnaire. We verified that all required assumptions and tests were met before fitting the PCA. The Kaiser–Meyer–Olkin (KMO) measure indicated suitability for conducting factor analysis, as its value was equal to 0.72 (Bartlett’s Test of Sphericity *p*-value < 0.001), and a positive determinant of our data matrix showed that we could move forward with fitting our PCA model. Three main components were identified in the analysis.

### 3.5. Principal Component Analysis (PCA) for the BAMBI

Table 5 displays the factor loadings of all 18 items across the three factors, along with h^2^ values representing the proportion of each variable’s variance accounted for by the principal components (i.e., the underlying latent constructs). Upon examining the items grouped within each factor, the three factors express the following variables: factor 1—Limited Refusals, factor 2—Food Refusal, and factor 3—Features of Autism.

## 4. Discussion

Considering the high prevalence of children with ASD and the possible serious complications of feeding difficulties, assessment and intervention should be performed using culturally and linguistically appropriate tools. The careful adaptation of the BAMBI has ensured that it is relevant, understandable, and valid for Greek-Cypriot parents, while retaining its validity and effectiveness.

The successful validation and cultural adaptation of the BAMBI for Greek-Cypriot children with ASD marks an important development in dealing with the feeding problems of this population while pointing out important features concerning significant differences among groups.

Statistically significant differences were found in BAMBI scores between parents of children diagnosed with ASD and parents of typically developing children in all three factors: Limited Refusal, Food Refusal, and Features of Autism. Higher scores by the ASD group for the prevalence of feeding problems agree with the existing literature, showing not only that issues occur often but also that they have crucial implications in terms of nutrition, development, and quality of life for children with ASD [21,26,27].

### 4.1. Reliability and Consistency

Foremost, the Greek version of the BAMBI maintained its strong psychometric properties, as evidenced in the original questionnaire, denoted by a Cronbach’s alpha of 0.755, showing demonstrated strong stability over time and robust internal coherence. The original three-factor structure of the BAMBI, including General Refusals, Refusing Food, and Autism-Related Features, was maintained and further established through PCA and CFA analyses, assuring this instrument’s construct validity in this new context [25]. High internal consistency and strong reliability are basic prerequisites for tools used at the clinical level to guarantee reliable data for interventions and planning.

### 4.2. Discriminatory Ability

Receiver Operating Characteristics analysis further validated the BAMBI-Gr’s effectiveness in discriminating between ASD children and typically developing ones: the Greek version of the BAMBI established optimal cut-off points that identify feeding problems. This reference can be used as practical guidelines for clinicians and researchers in the Greek-Cypriot community to detect difficulties in feeding early enough for targeted interventions, which may drastically improve the outcome for children with ASD.

Subsequently, it was noted that the results from the BAMBI-Gr validity studies concurred with those from the Turkish, Brazilian Portuguese, and Italian versions of the same instrument [21,26,27]. In all these studies, the internal consistency of the BAMBI was good to excellent, as indicated by alpha values. The alpha for the Greek version was 0.755, comparable to the Turkish version’s Cronbach alpha, 0.79, established by Meral & Fidan, 2014, while no questions scored above 0.7 for the Brazilian Portuguese version established by Castro et al., 2019 [26,27]. The Italian version, on the other hand, provided meaningful findings for both TD and ASD children, with alpha coefficients of 0.86 and 0.71, respectively [21]. These descriptive statistics demonstrate the tool’s capacity to measure the same constructs reliably across cultural contexts.

The test–retest reliability for the Greek version was very high, with an ICC of 0.999, reflecting that the tool provides stable results on repeated assessment. In this view, it would have been expected that this higher reliability resulted from the rigorous process of translation and back-translation performed with a detailed psychometric evaluation. Similarly high values of reliability were found for Turkish, Brazilian Portuguese, and Italian versions, which further confirmed the stability of the tool across repeated measures for these countries’ populations too [21,26,27].

Factor Structure Analysis across all cited versions confirmed the three-factor structure of the original BAMBI [25]: General Refusals, Refusing Food, and Autism-Related Features. This similarity of factor structure across different cultures lends considerable strength to the claim that the BAMBI framework is resilient in its assessment of the core features of mealtime behavior problems in children with ASD. Only the Greek version established this structure from the result of both PCA and CFA, while in the Turkish, Brazilian Portuguese, and Italian versions, it was through factor analyses only [21,26,27]. This outcome implies that, despite cultural variations, the underlying constructs measured by the BAMBI applied to all populations under study.

### 4.3. Sensitivity and Specificity

The Greek version of the BAMBI showed great sensitivity in distinguishing between children with ASD and typically developing children. ROC analysis provided the optimal cut-off points for detecting feeding problems and, thus, substantial information for identifying children who need intervention. The Turkish adaptation also showed good fit indices, with x^2^/sd = 3.6 and RMSEA = 0.09, confirming its capability for measuring mealtime behavior problems in a study by Meral & Fidan, 2014 [27]. Although neither the Brazilian Portuguese nor the Italian versions had available data on specificity and sensitivity, both were equally effective in identifying feeding difficulties [21,26].

Indeed, such variations in factor structures and model fit must be considered as reflecting different ways mealtime behaviors are experienced and reported across diverse cultural contexts. This comparison shows that instrument validation procedures are crucial, and they need to be performed in detail and with much sensitivity toward context for instruments like the BAMBI to detect the relevant behaviors as accurately as possible and yield reliable data for intervention and support in children with ASD.

### 4.4. Cultural Adaptation

A methodological process of translation and adaptation was used in all the previously described studies to ensure that the BAMBI was suitable for the population’s culture. In the Greek and Turkish studies, methods were followed precisely according to WHO guidelines involving bilingual experts and back-translation [27,28,29]. This probably helped attain the high reliability and validity scores obtained. Careful translation and validation procedures were carried out with the Brazilian Portuguese and Italian versions to ensure that the adapted tool was appropriate both linguistically and culturally [21,26]. These studies, taken together, therefore, demonstrate the strength of the BAMBI across cultures and, at the same time, highlight the issue of cultural adaptation in psychometric measures. Such procedures make clear that the need for cultural adaptation is a given in psychological assessment methods to retain integrity and applicability.

### 4.5. Clinical Implications

The BAMBI has the potential to serve as a valuable clinical tool for professionals working with children with ASD in Greek-Cypriot settings, offering a culturally adapted and psychometrically sound measure to better identify and address mealtime challenges. Given the possibility of an effective brief assessment of feeding difficulties offered by the BAMBI, early detection and intervention may enable them to avoid the increase in feeding problems. This inventory can be integrated into routine screenings and clinical assessments to avert feeding problems that, if left untreated, can influence the course of medical, developmental, and psychological outcomes. The implementation of the BAMBI may enhance the overall quality of care for children with ASD and promote better health and developmental outcomes.

### 4.6. Limitations and Directions for Future Study

While the results are beneficial, this study has some limitations. The sample was adequate for preliminary validation, but the sample size can be further expanded in future validation research with higher diversity and representation. It is important to acknowledge that the factor analysis was conducted on a moderately sized sample of 117 families, which is below the commonly recommended threshold of 200–300 participants for robust factor extraction. Although the analysis produced interpretable and stable results, a larger sample would strengthen confidence in the reliability of the three-factor structure. Future research with more diverse and representative samples will be essential to confirm the robustness of these findings. In particular, further validation of the tool in broader Greek-speaking populations is recommended to enhance its generalizability, especially among Greek-Cypriot parents. As outlined in our Introduction, the present study specifically aimed to validate the BAMBI for use by Greek-Cypriot parents of children with ASD and feeding difficulties. The recommendations for further research across diverse clinical presentations are intended as suggestions for future exploration beyond the scope of this study. In addition, longitudinal studies are warranted to evaluate the sustained efficacy of interventions informed by BAMBI assessments, thereby providing deeper insights into the tool’s clinical utility over time. Further investigation is also recommended to assess the tool’s applicability across diverse clinical presentations, including children with and without autism spectrum disorder (ASD), varying levels of ASD severity, and the presence or absence of feeding and swallowing disorders (FSDs). Such research would contribute to a more comprehensive understanding of the tool’s diagnostic and intervention-related relevance across heterogeneous populations.

## 5. Conclusions

The BAMBI-Gr demonstrates reliability, validity, and cultural suitability appropriate for assessing feeding problems in Greek-Cypriot children with ASD. This parent-completed questionnaire fills an important gap in existing resources, thus enabling better identification, assessment, and intervention. Successful cultural adaptation and validation of the tool support its clinical application in the Greek-Cypriot ASD community. Future research should continue extending the refinement and expansion of its use to keep it a cornerstone in assessing and addressing feeding difficulties in children with ASD.

## Figures and Tables

**Table 1:** Demographics of the sample.

	Parents Group of ASD Children (N = 42)	Parents Group of TD Children (N = 75)	
	M (SD)	M (SD)	*p*
Parents, Years of Age	40.59 (10.79)	39.78 (1074)	0.117 †
Age of father	41.75 (6.46)	39.63 (5.60)	0.069 †
Age of Mother	38.78 (4.90)	37.64 (6.64)	0.273 †
Children’s Years of Age	8.08 (3.60)	7.03 (3.54)	0.130 †

Abbreviations: TD, typically developing; ASD, Autism Spectrum Disorder; M, mean; SD, standard deviation; † independent sample *t*-test.

**Table 2:** Group comparisons of the BAMBI total score and its three factors.

	Parent Group of ASD Children (N = 42)	Parent Group of TD Children (N = 127)		
	Μ (SD)	Μ (SD)	t (115)	*p*
Limited Refusal Factor	51.92 (4.28)	20.16 (4.00)	4.672	<0.001
Food Refusal Factor	24.21 (5.28)	5.72 (1.33)	12.402	<0.0001
Features of Autism Factor	12.64 (4.51)	11.28 (2.63)	6.258	<0.001
BAMBI Total Score	51.33 (8.99)	37.16 (5.23)	10.787	<0.001

Abbreviations: TD, typically developing; ASD, Autism Spectrum Disorder; M, mean; SD, standard deviation; BAMBI, Brief Autism Mealtime Behavior Inventory.

**Table 3:** Group effects on the BAMBI total scores and its three factors.

	Parent Group of TD Children (N = 75)	Parent Group of ASD Level 1 Children (N = 11)	Parent Group of ASD Level 2 Children (N = 15)	Parent Group of ASD Level 3 Children (N = 16)		
	Μ (SD)	Μ (SD)	Μ (SD)	Μ (SD)	F(3, 113)	*p*
Limited Refusal	20.16 (4.00)	25.36 (2.87)	23.73 (7.11)	23.87 (4.71)	7.535	<0.001
Food Refusal	5.72 (1.33)	12.81 (3.89)	10.53 (5.22)	14.50 (3.38)	63.155	<0.001
Features of Autism	11.28 (2.63)	13.72 (1.74)	14.60 (3.29)	14.88 (2.60)	13.393	<0.001
BAMBI Total Score	37.16 (5.23)	51.91 (6.22)	48.86 (11.66)	53.25 (7.62)	40.354	<0.001

Abbreviations: TD, typically developing; ASD, Autism Spectrum Disorder; M, mean; SD, standard deviation; BAMBI, Brief Autism Mealtime Behavior Inventory.

**Table 4:** Pearson Correlations between BAMBI total score, BAMBI factors, and MCH-FS total score.

Variables	MCH-FS Total	BAMBI Total	Limited Refusal	Food Refusal	Features of Autism
MCH-FS Total	1.000	0.780 ***	0.647 ***	0.647 ***	0.586 ***
BAMBI Total	0.780 ***	1.000	—	—	—
Limited Refusal	0.647 ***	—	1.000	0.327	0.252
Food Refusal	0.647 ***	—	0.327	1.000	0.273
Features of Autism	0.586 ***	—	0.252	0.273	1.000

Footnote: BAMBI = Brief Autism Mealtime Behavior Inventory; MCH-FS = Montreal Children’s Hospital Feeding Scale. *** *p* < 0.001.

**Table 5:** Principal component analysis of the BAMBI questionnaire for the total sample.

Factor Items	Mean	Rotated Factor Loading	h^2^
**Factor 1—Limited Refusal**			
1. My child cries or screams during mealtimes.	1.49	**0.78**	0.74
2. My child turns his/her face or body away from food.	1.88	**0.66**	0.83
4. My child expels (spits out) food that he/she has eaten.	1.66	**0.82**	0.83
7. My child is disruptive during mealtimes (pushing/throwing utensils, food).	1.77	**0.73**	0.83
8. My child closes his/her mouth tightly when food is presented.	1.40	**0.61**	0.82
10. My child is willing to try new foods.	3.45	**0.54**	0.70
15. My child accepts or prefers a variety of foods.	4.10	**0.48**	0.65
**Factor 2—Food Refusal**			
11. My child dislikes certain foods and won’t eat them	2.93	**0.62**	0.43
12. My child refuses to eat foods that require a lot of chewing (e.g., eats only soft or pureed foods).	2.34	**0.54**	0.27
13. My child prefers the same foods at each meal.	2.83	**0.63**	0.5
14. My child prefers “crunchy” foods (e.g., snacks, crackers).	2.60	**0.49**	0.64
16. My child prefers to have food served in a particular way.	2.16	**0.54**	0.52
17. My child prefers only sweet foods (e.g., candy, sugary cereals).	1.66	**0.46**	0.73
18. My child prefers food prepared in a particular way (e.g., eats mostly fried foods, cold cereals, and raw vegetables).	1.85	**0.47**	0.61
**Factor 3—Features of Autism**			
3. My child remains seated at the table until the meal is finished.	4.14	**0.41**	0.50
5. My child is aggressive during mealtimes (hitting, kicking, scratching others).	1.36	**0.49**	0.66
6. My child displays self-injurious behavior during mealtimes (hitting self, biting self).	1.19	**0.72**	0.54
9. My child is flexible about mealtime routines (e.g., times for meals, seating arrangements, place settings).	3.39	**0.62**	0.73

Factor loadings above 0.40 are marked in bold, and the highest value is underlined.

## Data Availability

Due to ethical restrictions, the data generated during or analyzed in the current study are not publicly available. For consideration, all data queries and requests should be submitted to the corresponding author, Andri Papaleontiou.

## Abbreviations

Below is a list of abbreviations used in this manuscript:

APC	Article Processing Charge
ASD	Autism Spectrum Disorder
BAMBI	Brief Autism Behavior Inventory
CFI	Comparative Fit Index
CVI	Content Validity Index
FSD	Feeding Swallowing Disorder
M	Mean
PCA	Principal Component Analysis
RMSEA	Root Mean Square Error of Approximation
ROC	Receiver Operating Curve
SD	Standard Deviation
TD	Typical Developing
TLI	Tucker–Lewis Index
WHO	World Health Organization
